# In vivo wear of CAD-CAM composite versus lithium disilicate full coverage first-molar restorations: a pilot study over 2 years

**DOI:** 10.1007/s00784-020-03294-5

**Published:** 2020-05-12

**Authors:** Jan-Frederik Güth, Kurt Erdelt, Christine Keul, Gintare Burian, Josef Schweiger, Daniel Edelhoff

**Affiliations:** Department of Prosthetic Dentistry, University Hospital, LMU Munich, Goethestrasse 70, 80336 Munich, Germany

**Keywords:** Abrasion, Wear, Dental materials, CAD/CAM composite, Lithium disilicate, Wear behavior

## Abstract

**Objectives:**

To present a digital approach to measure and compare material wear behavior of antagonistic first molar restorations made of an experimental CAD/CAM composite (COMP) and lithium disilicate ceramic (LS2) in patients with reconstructed vertical dimension of occlusion (VDO) after generalized hard tissue loss.

**Methods:**

A total of 12 patients underwent complete full jaw rehabilitation with full occlusal coverage restorations made either of COMP or LS2. The first molar restorations (*n* = 48) were chosen for wear examination. At annual recall appointments, polyether impressions were taken, and resulting plaster casts were digitalized using a laboratory scanner. Mean observation period was 371 days for first and 769 days for second year. The resulting 96 datasets were analyzed by superimposition of 3-D datasets using an iterative best-fit method. Based on the superimposition data, the wear rates of the occlusal contact areas (OCAs) were calculated.

**Results:**

For antagonistic restorations made of COMP, the average wear rate was 24.8 ± 13.3 μm/month, while for LS2, it was 9.5 ± 4.3 μm/month in first year, with significant differences (*p* < 0.0001) between the materials. In second year, monthly wear rates decreased significantly for both materials: COMP (16.2 ± 10.7 μm/month) and LS2 (5.5 ± 3.3 μm/month). Statistical comparison between wear time showed significant differences for both materials: COMP *p* < 0.037 and LS2 *p* < 0.001. A logarithmic fit (COMP *R*^2^ = 0.081; LS2 *R*^2^ = 0.038) of the data was calculated to estimate the wear progression.

**Significance:**

In patients with reconstructed VDO, restorations made of LS2 show a more stable wear behavior than ones out of experimental CAD/CAM composite. In cases of complete rehabilitation, load bearing CAD/CAM-composite restorations should be critically considered for application due to their occlusal wear behavior. However, when choosing a restorative material, not only the functional occlusal stability should be taken into account but also the prospect of minimally invasive treatment with maximum preservation of natural tooth structures.

## Introduction

Tooth wear is a condition of growing concern these days. Loss of dental hard tissue has a multifactorial etiology. Basically, there are three main reasons which lead to worn dentition (erosion/bio corrosion, abrasion, and attrition), and previous studies showed that these wear mechanisms show mutual interactions [1, 2]. Possible consequences of an accelerated loss of hard tissue can be a reduced vertical dimension of occlusion (VDO), an increased tooth sensitivity, and changes in esthetics and function. The rising prevalence and incidence of this complex condition forces dentists to develop new strategies for diagnosis, patient monitoring, and treatment in order to react as early and effectively as possible, using minimal invasive methods [3, 4].

CAD/CAM polymers, also termed high-performance polymers (HPP), have been introduced to the market as an alternative to ceramics [5, 6]. Polymerized following industrial standards and processed by subtractive methods using CAD/CAM technology, the mechanical properties of these materials are considered to be superior to those of direct polymers [7]. However, the possible applications of CAD/CAM polymers clearly depend on their individual chemical composition, as the individual parameters significantly influence their mechanical properties [8]. Today, these CAD/CAM polymers on the basis of highly cross-linked PMMA resins or filled composites are offered by numerous manufacturers. They attract interest in different fields in dentistry and allow numerous novel treatment options [9, 10].

Currently, CAD/CAM polymers on the basis on PMMA are used as long-term temporary restorations during extended pre-treatment phases of up to 2 years [11]. Their material properties allow ultra-thin restoration designs, which dispense extensive tooth preparation and lead to significant dental hard tissue preservation [12, 13]. However, the prospective transition to definitive ceramic restorations requires the clinician to prepare the teeth to ensure an adequate occlusal thickness and an appropriate edge design for the restorations [14]. Inevitably, this may lead to an additional loss of tooth structure.

Keeping this in mind, CAD/CAM polymers on the basis of highly filled composites might constitute a new definitive treatment approach without or with only minimal hard tissue loss. These polymers harbor favorable grinding/milling properties, and due to low modulus of elasticity, it results in higher edge stability, so that these polymers can be used in thinner designs than ceramic materials [10]. Some manufacturers have been offering similar materials for several years now and recommend their application as definitive restorations under clinical conditions. So far, no clinical data have been available on the long-term behavior of these restorations. The main limitation is that clinical research presents many challenges as patient recruitment, funding, and extended time to accumulate some reliable data on clinical restoration changes. Many different 3-D measuring techniques were used in the past, to provide quantitative data on dental materials wear [15, 16]. These comparable methods were used in previous studies mostly reporting on the wear of single posterior composite crowns to be around 40 μm/year [17, 18]. The difference to the present study is that single crowns located within a tooth row were evaluated, but not a full arch reconstruction was conducted out of composite material. It can be assumed that in the previous studies the single crowns were protected by adjacent structures, which could result in comparable wear to adjacent structures. Whereas when a full mouth reconstruction is carried out, the wear behavior of the CAD/CAM polymers might be different especially if, as in the present study, they are to be used to maintain the reconstructed vertical dimension of occlusion (VDO).

The purpose of this clinical pilot study was (1) to present, apply, and evaluate a digital method for measuring wear in a clinical setting and (2) to assess the wear behavior of two restorative materials in patients with a reconstructed VDO after a generalized loss of tooth structure. In this study, an experimental CAD/CAM composite was compared with a lithium disilicate ceramic regarding longitudinal abrasion characteristic over 2 years. The null hypothesis of the study was that the restorations made of the experimental CAD/CAM composite exhibit similar wear rates as restorations made of lithium disilicate ceramic.

## Materials and methods

### Patients

The study was performed in accordance with the Code of Ethics of the World Medical Association (Declaration of Helsinki) after approval by the Ethics Committee of the university hospital of LMU Munich (012-12; 541-12).

A total of 12 (7 males, 5 females; mean age, 36.3 ± 9.4 years) patients with changes in the vertical dimension of occlusion (VDO) due to loss of hard tissues were included in the study. In all patients, restoring the vertical dimension of occlusion with full arch antagonistic restorations in both jaws was indicated (no-prep occlusal veneers, partial crowns, or full crowns). Canine-guided occlusion through rehabilitation was achieved in every patient. The following inclusion criteria for study participation were defined:

- Age above 18 years and under 70 years.
- Appropriate, at least average oral hygiene.
- Extended decrease of the vertical dimension of occlusion (VDO) due to attritional, abrasive, erosive, or pathological damage to the tooth structure.
- Indication for a minimum of 12 restorations in antagonistic jaws.
- Healthy/treated periodontal tissues (at most grade 1 tooth mobility).
- Pregnant and breastfeeding women were excluded from the study.

All patients participating in the study were informed about the background of the study and the risks associated with it and gave their informed consent.

The patients were divided into two groups:

1. Group COMP included 6 patients, who received adhesively bonded CAD/CAM restorations (*n* = 168) made of experimental, industrially polymerized composite blocks.
2. Group LS2 consisted of 6 patients, who received monolithic ceramic restorations (*n* = 168), as control group.

**Fig. 1:**
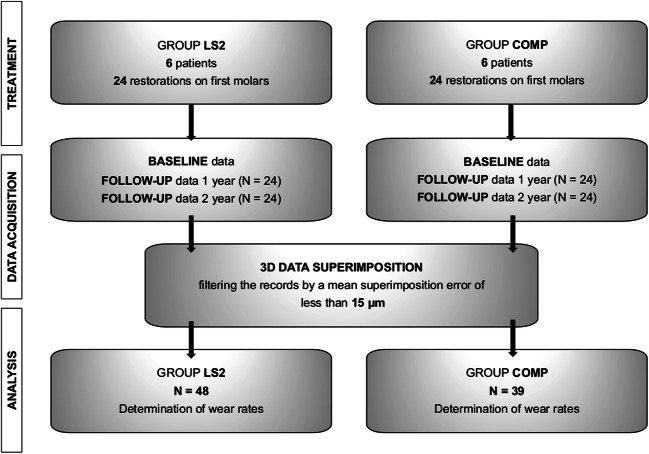
Overview of the study design and procedure

The wear rate was determined based on the first molar restorations (COMP *n* = 24; LS2 *n* = 24) in the maxilla and mandible. Measurements were performed after first and second year for each first molar which resulted in 96 post control datasets. An overview of the study process is shown in Fig. 1.

### Treatment and laboratory procedures

The experimental composite material (Ivoclar Vivadent, Schaan, Liechtenstein) consisted of 22% *V*_f_ matrix (dimethacrylate) and 78% *V*_f_ filler (barium glass fillers, 15%; ytterbium trifluoride, 9%; mixed oxides, 44%; silicon oxides, 3%; copolymers, 7%). The material used in this study exhibited the manufacturer’s properties which were as follows: flexural strength = 167 MPa, modulus of elasticity = 11.4 GPa, Vickers hardness = 915 MPa, and water absorption after 7 days = 28 μg/mm^3^.

Mechanical properties of used lithium disilicate ceramic (IPS e.max Press, Ivoclar Vivadent, Schaan, Liechtenstein) according to manufacturer are as follows: flexural strength = 400 ± 40 MPa, modulus of elasticity = 95 ± 5 GPa, and Vickers hardness = 5900 ± 100 MPa (https://www.ivoclarvivadent.com/en/p/laboratory-professional/products/all-ceramics/ips-emax-technicians/ips-emax-press).

The clinical procedure in both groups was conducted corresponding to the state of the art in current adhesive (minimally invasive) restorations. Necessary core build-ups were made with direct low viscosity (Tetric EvoFlow, Ivoclar Vivadent, Schaan, Liechtenstein) and/or high-viscosity composites (Tetric EvoCeram, Ivoclar Vivadent, Schaan, Liechtenstein) and a multi-step adhesive system (Syntac, Ivoclar Vivadent, Schaan, Liechtenstein). Impressions were taken with polyether (Permadyne/Impregum Penta, 3 M, Seefeld, Germany) by an individualized Rimlock tray. Fabrication of the restorations was completed in a dental laboratory by an experienced dental technician.

COMP restorations were designed and manufactured using the Cerec system (CEREC InLab V3.86, Dentsply Sirona, Bensheim, Germany), with the following settings: proximal contacts strength = 75 μm, occlusal contact strength = 25 μm, and adhesive gap = 20 μm. Before placing the composite restorations, the inner surfaces were prepared using modified Rocatec procedure (Rocatec soft 30 μm; 1 bar; nozzle distance, 2 cm; 5s blast time per unit) and conditioned with Monobond Plus (Ivoclar Vivadent, Schaan, Liechtenstein).

LS2 restorations were fabricated using the press technique. The inner surfaces of the lithium disilicate restorations were etched with 5% hydrofluoric acid (IPS Ceramic Etching Gel, Ivoclar Vivadent, Schaan, Liechtenstein) for 20 s, rinsed with air/water spray for 60 s, and cleaned in ultrasonic bath for next 60 s. Then, silan coupling agent as part of Monobond Plus (Ivoclar Vivadent, Schaan, Liechtenstein) was applied for 60 s.

Adhesive bonding in both groups was performed with Total Etch & Rinse technique using Syntac (Ivoclar Vivadent, Schaan, Liechtenstein) in combination with the Variolink II (Ivoclar Vivadent, Schaan, Liechtenstein) and light curing, following the manufacturer’s instructions. If necessary, occlusal adjustments in static and dynamic occlusion were performed with ball-shaped diamond finishing bur (8801 314 018, Komet Dental, Lemgo, Germany) and water spray application. Finally, the adjusted occlusal areas were polished by adequate polishing sets (Composite: Set 4312A, Ceramic: 4313B, Komet Dental, Lemgo, Germany).

### Baseline and follow-up

To investigate the wear behavior of the restorations in both groups, dental impressions with a polyether impression material (Impregum Penta, 3M Espe, Seefeld, Germany) were taken after adhesive bonding and occlusal adjustments of the restorations (baseline). The impressions were poured between 24 and 48 h with type IV dental stone (Plurastone, Pluradent, Offenbach, Germany). The resulting plaster casts were stored at room temperature 21 °C ± 1 °C. All gypsum models were scanned with a laboratory scanner (D810, 3Shape, Copenhagen, Denmark). All follow-up recalls were performed by the same experienced clinician at approximate 12-month interval after clinical loading. Mean observation period in both groups was 371 ± 106 days (first year) and 769 ± 102 days (second year).

### Processing of datasets

**Fig. 2:**
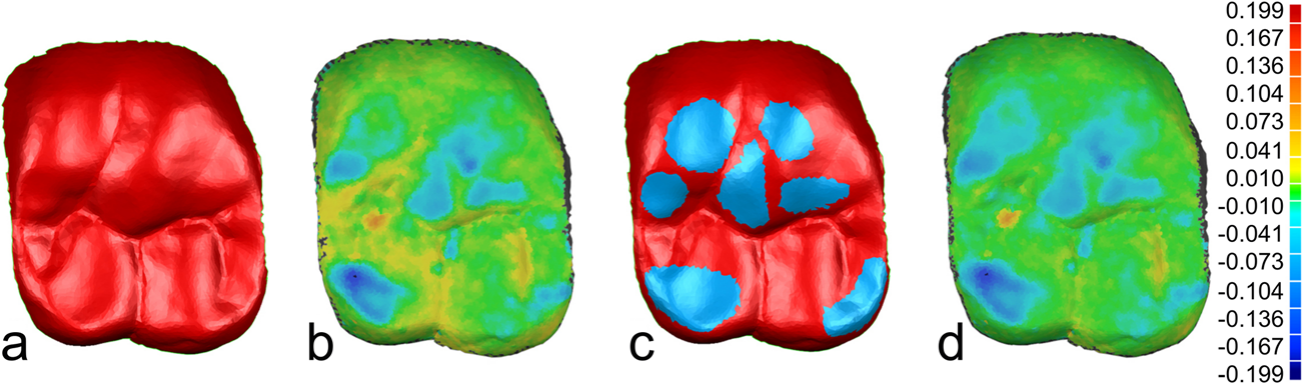
Example of the iterative approach to the overlay of baseline and follow-up datasets: **a** overlay over the entire occlusal surface; **b** color-coded representation of the differences between baseline and follow-up data after the first overlay; **c** exclusion of areas with antagonistic wear, to achieve fitting of the areas that are not changing; **d** color-coded representation of the differences between baseline and follow-up data after superimposing and exclusion of the worn surfaces. This procedure was iterated until the overlay error no longer changed

The resulting STL datasets at baseline and follow-up recalls were imported into the Geomagic Qualify 2012 surface matching analytical software (Geomagic Inc., Morrisville, NC, USA). As a first step, the individual restored first molars of the digital models were isolated and stored as separate datasets in order to facilitate a restoration-related analysis. The data points below the tooth equator were eliminated. Subsequently, the recall data were superimposed with the baseline data, initially highlighting the entire restauration surface of the reference dataset and the follow-up dataset using a best-fit method. The result of this overlay was visually evaluated and the average overlay error determined. Next best-fit alignment was conducted only over those surfaces in which the deviation was less than the overlay error. This procedure was iterated until the overlay error no longer decreased. Only datasets with an overlay error of less than 15 μm were further processed (COMP *n* = 39; L2S *n* = 48). Figure 2 illustrates an example of the procedure. The error of the superimposition was documented for each specimen individually.

**Fig. 3:**
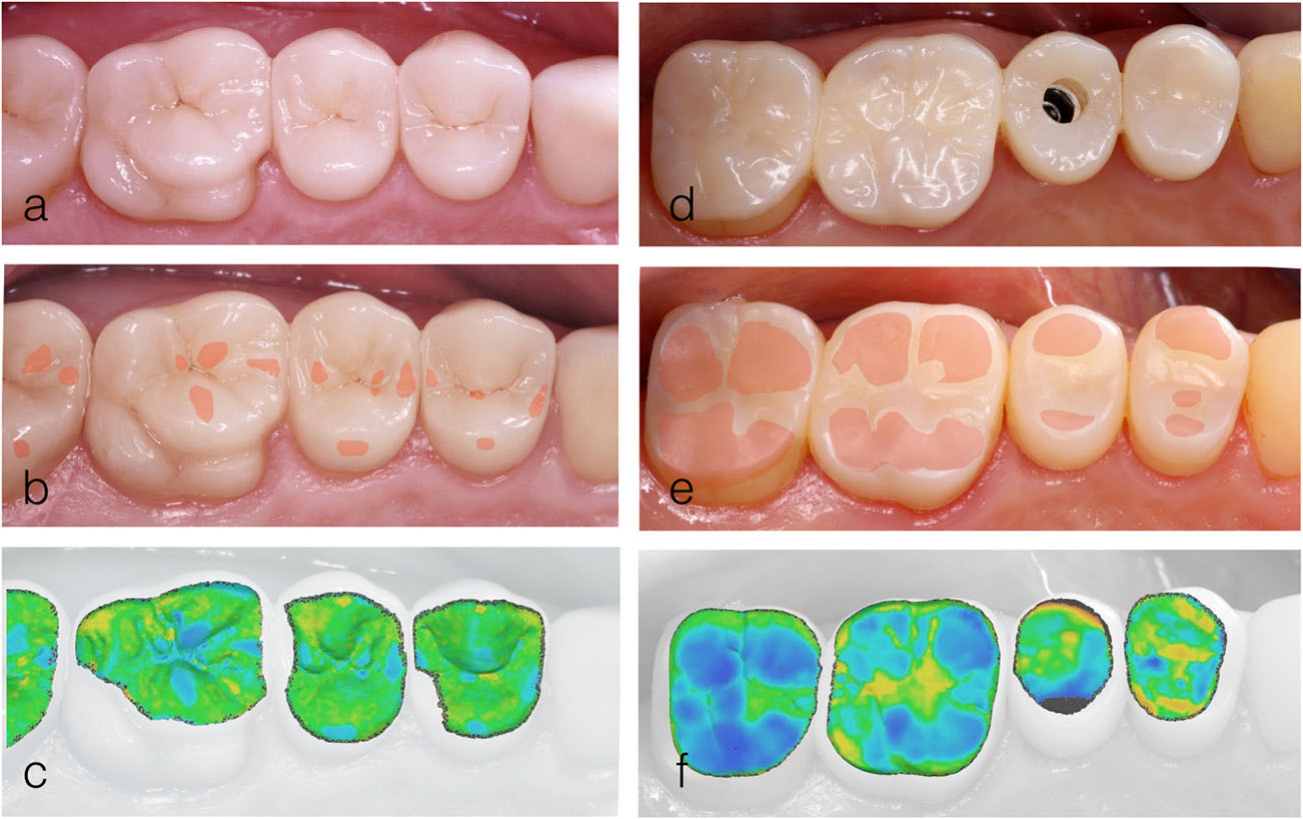
Color-coded representation of the abrasion behavior: **a** clinical photograph taken at baseline after restoration with lithium disilicate ceramics, **b** clinical photograph taken at the 24-month follow-up (worn surfaces were later marked in red), **c** color-coded representation of the deviations following data overlay, **d** clinical photograph at baseline after restoration with an experimental CAD/CAM composite, **e** clinical photograph taken at the 12-month follow-up (worn surfaces were later marked in red)

This iterative approach allowed to delineate those areas of the restorations that showed signs of wear. After completion of the superimposition, the differences between the datasets were visualized by color-coded pictures which reproduced wear caused by the antagonist restoration. Only areas in which wear could be detected (blue color coding) were selected for further wear analysis. The distance data was exported and stored as individual result files (.csv). Figure 3 shows an example of the wear behavior based on color-coded representation.

### Wear evaluation

The resulted files were imported into a statistics program SPSS (version 25, IBM, Armonk, NY, USA) and prepared for further analysis of wear. Subsequently, the average wear depth and the maximum wear depth were calculated perpendicular to the surface of the restoration. To ensure comparability of data in spite of different times in situ, wear was calculated by dividing the values by the number of wear days. Afterwards, the average wear rate per month and the average maximum wear rate per month were determined for the material groups. Furthermore, the data were analyzed for significant differences between the groups of materials (Mann-Whitney *U* test). The *p* value was set at 0.05.

## Results

### Superimposition error results

**Fig. 4:**
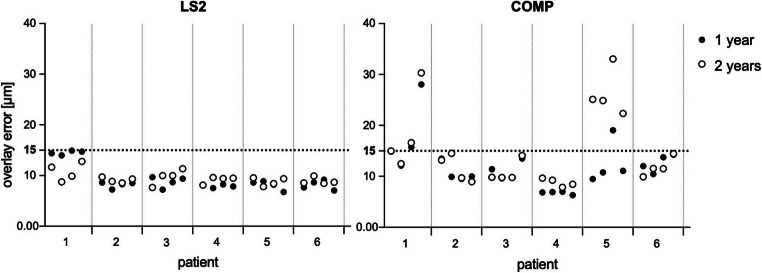
Overlay error of each molar specimen in accordance with individual patients

A prerequisite for further analysis was a superimposition error between baseline and follow-up datasets less than 15 μm after data overlay (Fig. 4). It turned out that the follow-up data for the experimental CAD/CAM composite caused larger superimposition errors in overlaying process than lithium disilicate. The group COMP exhibited a mean overlay error of 11.9 ± 4 μm after first-year follow-up, compared with 9.3 ± 2 μm in the group LS2. Furthermore, second-year follow-up datasets results confirmed increased superimposition errors in both groups. Group COMP showed an overlay error of 14.6 ± 7 μm, which was significantly higher in relation to first-year values. As a consequence, more datasets had to be excluded in second year. Group LS2 remained near constant with mean superimposition error of 9.4 ± 1 μm. Filtering out the datasets with overlay errors less than 15 μm, the number of analyzed records was reduced to 39 in group COMP. All lithium disilicate ceramics fulfilled these criteria, and 48 records were included for further analysis.

### Average wear rates per month

The values for the wear rates per month in first and second year after placement for COMP and L2S are shown in Table 1. Table 2 shows results of wear rates per year. The Kolmogorov-Smirnov test showed that no normal distribution of values was present; thus, the Mann-Whitney *U* test was used for statistical comparison between materials and wear time.

**Table 1:** Average wear rates per month [μm]

	Time	N	Mean	SD	Median	95% CI	IQR
LS2	1 year	24	9.46	4.31	8.67	7.63/11.28	4.56
	2 years	24	5.47	3.29	4.44	4.08/6.86	2.84
COMP	1 year	21	24.76	13.32	24.92	18.69/30.82	24.50
	2 years	18	16.23	10.72	13.61	10.89/21.56	12.97

**Table 2:** Mean wear rates per year [μm]

	Time	N	Mean	SD
LS2	1 year	24	113.52	51.72
	2 years	24	65.64	39.48
COMP	1 year	21	297.12	159.84
	2 years	18	194.76	128.64

**Fig. 5:**
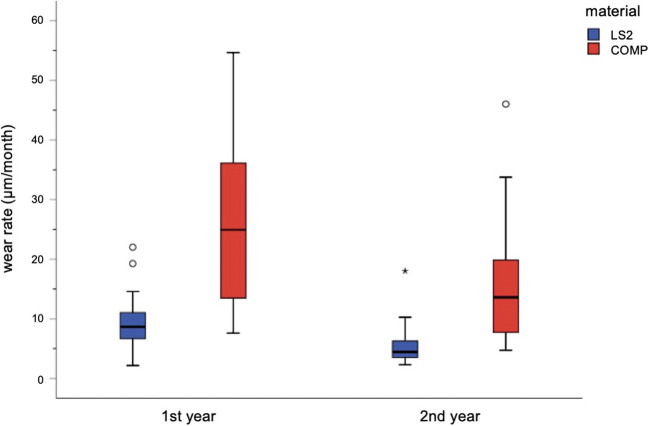
Mean wear rates (μm/month). Boxplots illustrate median and IQR values. Circles represent the outliers

Analyzing first-year data showed statistically significant differences (*p* < 0.001) between COMP (24.8 ± 13.3 μm/month) and LS2 (9.5 ± 4.3 μm/month). Second-year data showed decreased wear rates per month for both materials, still with significant differences (*p* < 0.001): COMP (16.2 ± 10.7 μm/month) and LS2 (5.5 ± 3.3 μm/month). Statistical comparison of wear between first and second year showed significant differences for both materials: COMP *p* < 0.037 and LS2 *p* < 0.001. The results are shown in Fig. 5.

### Maximum wear rates per month

**Fig. 6:**
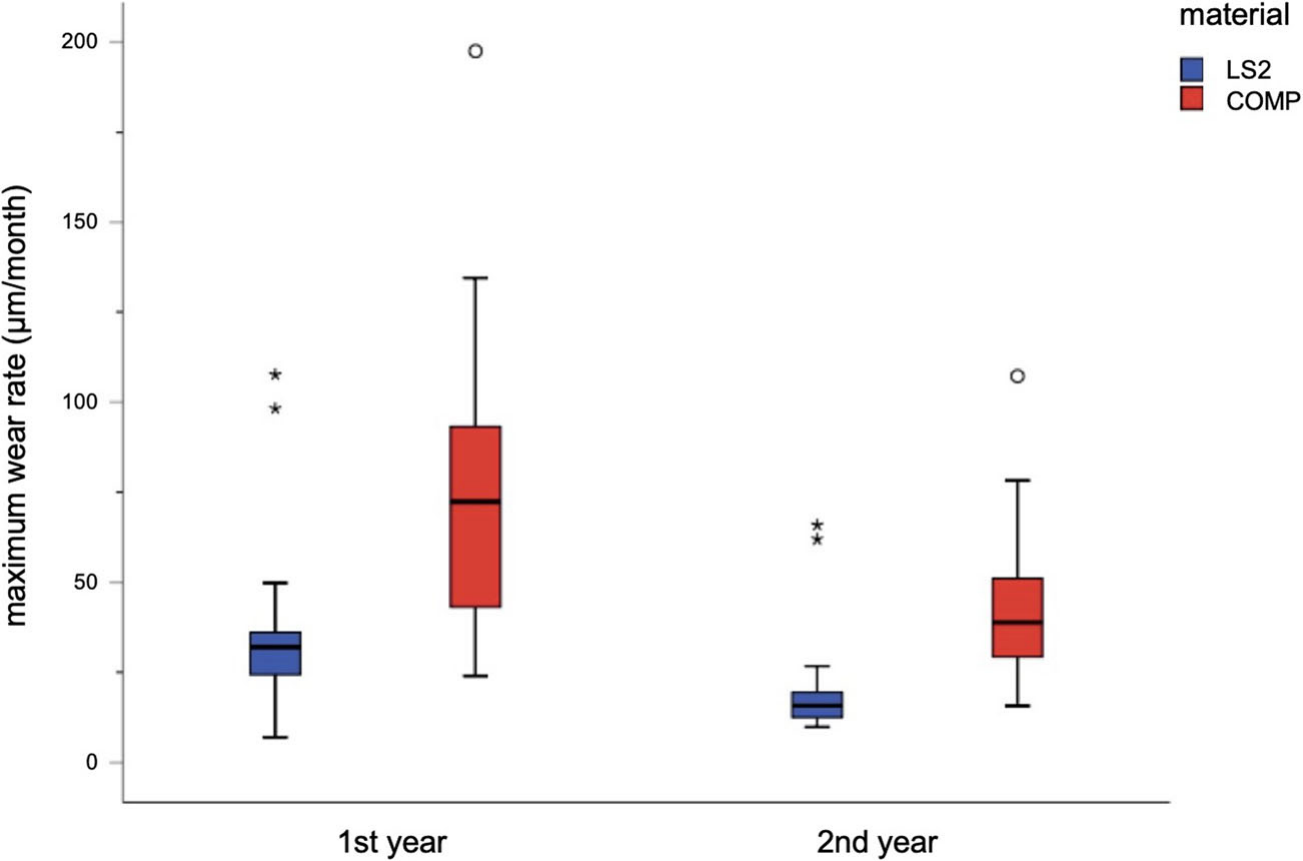
Maximum wear rates (μm/month). Boxplots illustrate median and IQR values. Circles represent the outliers

The values for the maximum depth of wear per month in first and second year are shown in Table 3 and Fig. 6. The Kolmogorov-Smirnov test showed that no normal distribution of values was present; thus, the Mann-Whitney *U* test was used for statistical comparison between materials and wear time. The average maximum wear rate across all restorations made of the experimental CAD/CAM composite was 76 ± 42.9 μm/month which was significantly different (*p* < 0.001) from the average maximum wear rates of 36.1 ± 22.6 μm/month for restorations made of lithium disilicate ceramics in first year. In second year, exhibited maximum wear rates decreased for both materials: COMP 45 ± 23.3 μm/month and LS2 19.9 ± 14.3 μm/month; these results still showed significant differences (*p* < 0.001). Figure 6 shows the corresponding box plots.

**Table 3:** Maximum wear rates per month [μm]

	Time	N	Mean	SD	Median	95% CI	IQR
LS2	1 year	24	36.13	22.55	31.99	26.61/45.65	12.09
	2 years	24	19.88	14.33	15.66	13.83/25.93	7.24
COMP	1 year	21	76.03	42.91	72.32	56.50/95.56	58.37
	2 years	18	45.06	23.27	38.86	33.49/56.63	23.50

### Wear in time axis

**Fig. 7:**
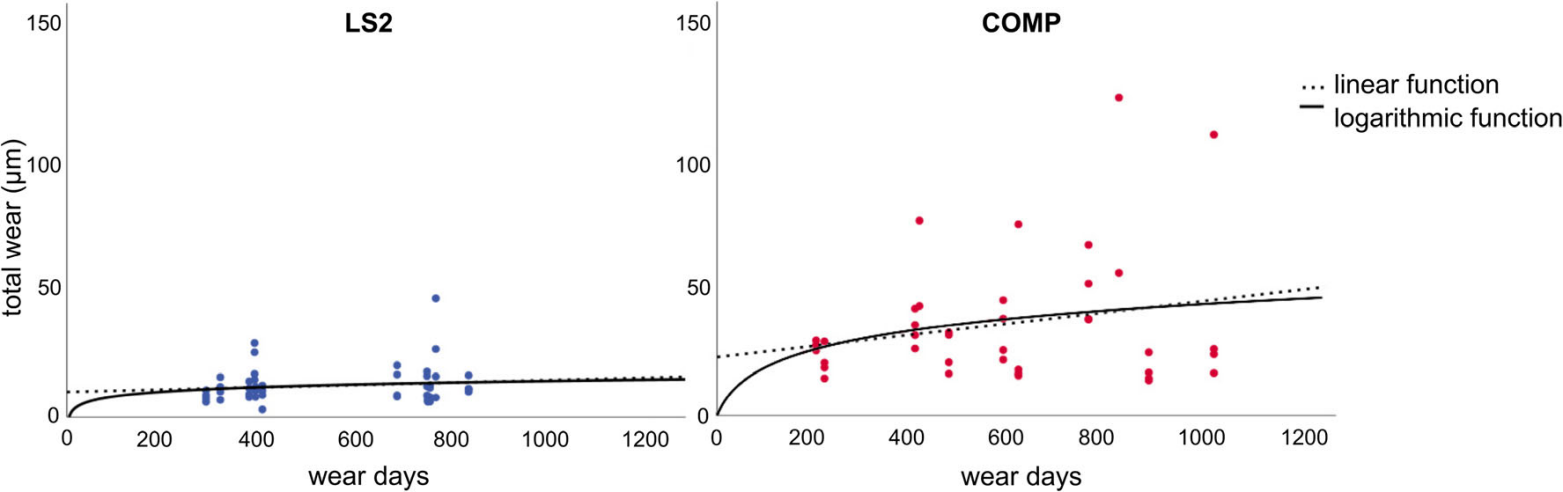
Wear over time axis of each individual specimen

The progress of total wear over time for each specimen is shown in Fig. 7. The following graph demonstrates continuous increase of wear for both materials. Based on the previous statements, the statistically significant decrease of wear rates per month over time confirms that abrasion shows a time dependency. The highest overall loss of restorative material occurred during the first year of use, whereas between the first and second year, the amount of wear decreases. Curve fitting was calculated and adjusted with SPSS to analyze time dependence of wear.

Two assumptions were made for curve fitting: (1) At starting point, when restorations were placed, no wear had occurred yet. (2) The total wear increases with time. Based on these two assumptions, only linear and logarithmic functions are possible. However, a linear function does not fulfill assumption one, because it does not cross the *y*-axis at zero. Therefore, linear increase of wear rates must be rejected. Logarithmic function (COMP *R*^2^ = 0.081; L2S *R*^2^ = 0.038) showed the best fit to these data points, as the function starts almost at zero and increases continuously.

## Discussion

The present clinical pilot study compared the wear behavior of antagonistic monolithic restorations made of two materials: experimental CAD/CAM composites and lithium disilicate ceramics. To our best knowledge, the present study is the first so far to compare wear of CAD/CAM composite versus CAD/CAM composite with lithium disilicate versus lithium disilicate in vivo conditions [19]. The results showed significant differences in wear progress between these two materials in patients that received full mouth rehabilitation. Restorations made of the experimental CAD/CAM composite exhibited higher wear rates than those made of lithium disilicate ceramics. The null hypothesis must therefore be rejected.

The average wear rates per month were higher in first year compared with the follow-up values in both groups. It should be taken into consideration that these wear rates will decrease every year as a possible consequence of the formation of the occlusion wear facets.

While the wear area increases, the applied forces are distributed onto a larger area. This reduces the forces per area and might the reason that wear rates are highest initially after placing the restorations, as restorations are adjusting to each other. Longitudinal studies are still needed to confirm our expectations on this wear behavior in vivo for extended follow-up intervals. As well, the possibility of non-contact area wear of composites, that is caused by failure of composite components, cannot be refused [20]. This might influence the accuracy of the superimposition and might even lead to higher overall wear in the composite group, which even more supports the findings of this study.

Quantitative wear measurement acquired in this study’s clinical setting was assessed using a new iterative approach. Measurements were performed on entire occlusal surface on every first molar, using plaster casts after conventional impression. To minimize potential errors of this workflow, the overlay and analysis were performed for each molar individually, to gain a certain independence of adjacent structures. This made it possible to eliminate at least the influence of overall distortions of impressions and manufacture of plaster casts that could influence the results [21]. In addition, the superimposition process was iterated until the overlay error was no longer changed by further superimposition. In this way, the best fit of baseline and follow-up data over the areas that had not been exposed to any antagonistic wear could be achieved.

For the quantitative measurement of vertical height loss of antagonistic restorations, mean superimposition error of 15 μm was determined as the standard deviation error between the data records. In vitro studies, the standard deviation of superimposition has been described to be between 5 and 10 μm [22]. Against that data, capturing under clinical conditions seems to be more error prone and present higher variations; therefore, higher standard deviations up to 15 μm had to be accepted. On the other side, data bellow tolerance of 15 μm were excluded in this study to receive most reliable data. Based on individual bite forces and masticatory movements, every investigated specimen exhibited different pattern of abrasion. DeLong et.al reported that the estimation of superimposition for samples of clinical studies usually fluctuate from 10 to 20 μm per point; they considered the superimposition of less than 10 μm to be an excellent fit, whereas a value of more than 50 μm indicates a poor fit [15]. This goes in line with other clinical investigations in measuring wear, where workflow inaccuracy in the range of 15–20 μm was considered acceptable [18, 23–26]. Schmid-Schwap et al. [27] even set the limit at 30 μm for standard deviation/workflow inaccuracies for molars as reasonable, as they are more difficult to superimpose. However, this was stated for the wear of methacrylate artificial teeth. In presented study, composite restorations showed higher superimposition errors which may be caused by non-antagonistic wear which is more pronounced on composite than lithium disilicate ceramic [28].

Wear can be quantified using depth, area, and volume. The connection and correlation of these parameters are explained excellent by DeLong et al. [29]. However, the methodology of wear measurements under clinical conditions is currently intensively discussed [30–34]. In this clinical study, the parameter of vertical height loss measured perpendicular to working surface was applied. The calculation of volume changes under clinical conditions seems to be very error prone. Most critical point is to define area for volume calculation, between exposed and non-exposed area of wear. In contrast, the reproducibility and comparability of these measurements were restricted, due to high failure rate on assessing thin margins and different surface areas, what made this data unusable. Furthermore, previous studies already elucidated the advantage of measuring vertical height loss compared with volumetric wear measurement. The main advantage is the possibility of a direct quantification of wear and eliminating the influence of surface size [27, 31]. A combination of vertical height loss and surfaces measurement was desirable; however, surface measurement underlay the same trouble as volumetric measurements.

According to the biomimetics concept, the wear behavior of dental restorations should ideally resemble physiologic tooth enamel. However, clinical data on the wear behavior of natural teeth is rare and varies widely. In the few existing studies, the wear rates for natural enamel were found to be about 10–40 μm/year [35–37]. The parameter that was comparable with the measurements reported in the literature seems to be the mean wear rate per year. However, the results of clinical trials showed considerable fluctuations [30]. The wear rates differed significantly between different research groups; Etman et al. [38] stated wear of 148 μm after first year for lithium disilicate glass-ceramic posterior crowns, whereas Kramer et al. [39] determined it as 78 μm after 4 year for ceramic inlays made of lithium disilicate. Only very few studies investigated wear of posterior composite crowns in vivo and reported wear to be around 40 μm/year, which is considerably lower than values found in this study [17, 18]. Although there is an abundance of clinical data on the wear characteristics of direct composite restorations, literature shows a considerable variation in results from 50 to 200 μm per year [30, 40, 41]. It must be taken into account that these studies compared wear rates with opposing enamel as an antagonist. Besides that, direct composite resin restorations of class I/II are protected by enamel which limits the conclusions drawn in regard of biomechanical loading. Additionally, not only antagonistic situation but also the clinical environment in which the restorations are placed seems to play a role for wear rates, and it is clear that wear rates might differ for single restoration against different antagonists. In the present study, the patients received full mouth rehabilitation using either CAD/CAM composite or lithium disilicate. In this clinical constellation, restorations are not protected by adjacent structures like enamel or crowns from other materials with lower wear rates. This means that the restorations in this study are subjected to complete bite forces and have to carry the full occlusal load either by opposing composite or ceramic. There is still no reliable data on wear rates in clinical cases of posterior restorations in cases of complete rehabilitation. Therefore, it is challenging to validate the credibility of our results. In this pilot study, wear was measured only on first molars. Further analysis applying the same methodology would also be considerable to make a distinction between premolars and molars, as some studies confirmed wear was more pronounced on molars than premolars [42, 43].

The analysis of the wear behavior of restorative materials and tooth structure in clinical cases poses two main problems for scientists and clinicians: taking exact and reproducible impressions and finding an adequate valid and reliable method for analysis of wear. Distortions or impression tolerances are two among other potential factors impairing the resulting cast quality [21, 44]. Moreover, the scanning process itself is prone to further inaccuracies, affecting the measurement results [45]. To avoid errors due to plaster cast fabrication, it is possible to scan impressions directly; however, undercuts and steep tooth geometries seem to limit this approach [46]. Also from the point of accuracy, there seems no significant advantage due to digitizing a conventional impression directly compared using the poured plaster model [47]. A considerable change of this step in the workflow would be the use of intraoral scanner for digital impressions. However, in vivo studies validated that conventional precision impression materials, like polyether used in this study, still show higher precision for full-dental arch impression compared with current intraoral scanners systems [47, 48]. To exclude any influence of global distortion and inaccuracies of data acquisition, the present analysis is based on single tooth areas after sectioning the virtual dataset. Against this background, an application of intraoral scanner seems to be reasonable for further studies. Another limitation of the present pilot study was the relatively small cohort size. However, after an accumulated number of 87 numbers of datasets, significant differences between the groups of materials could already be detected. Also, it can be presumed that our patients showed parafunctional behavior after altering the VDO, which could lead to increased wear of material during the first year. But so far, there is no evidence based on well-controlled clinical trials regarding this correlation [49]. However, based on the results for both types of restorations and assuming functionally active patients, an additional protective splint (night guard) may be recommended to be used at least overnight to prevent repeated early loss of vertical dimension.

To estimate the annual loss of vertical dimension, the average wear rates need be doubled because wear takes place in both jaws. Nevertheless, potential dental compensation has to be considered when this conclusion is drawn [50]. This would mean a total loss of height of 49.5 μm/month in the posterior region for restorations made of the experimental CAD/CAM composite and 18.9 μm/month for restorations made of lithium disilicate ceramics in first year after placement. Based on the assumption of logarithmic wear model, this trend might be reduced over following years in function. However, this hypothesis has to be proven in further studies. Seen from this point of view, restorations made of lithium disilicate ceramic seem to offer a more stable prognosis in terms of wear and prevention in the long run.

On the other hand, CAD/CAM composite restorations also have some advantages. These include the minimal invasiveness due to the better properties of CAD/CAM composite and higher flexibility of the material compared with ceramics [51]. The characteristics of the polymeric material and the superior edge stability facilitate procedures with very thin layers of the material and with little depth of preparation margins—or even without any preparation at all—which seems advantageous in terms of maximum tooth conservation. Moreover, CAD/CAM composite restorations have been associated with a more favorable wear behavior on the antagonistic enamel, which seen from a biomimetic point of view should be preferable as tooth structures will be preserved [52].

In summary, it can be concluded that in patients with a generalized loss of tooth substance, partial coverage restorations made of monolithic lithium disilicate ceramics to reconstruct the VDO showed lower wear rates than similar restorations made of an experimental CAD/CAM composite. Further studies are necessary to show whether these results are also valid for different clinical environments and settings, as well as for natural enamel antagonists.

## Conclusion

Within the limitations of the present experiment, the following could be concluded. (1) COMP wore significant more than L2S (16.2 ± 10.7 μm/month vs 5.5 ± 3.3 μm/month, *p* = 0.001) after 2 years, and (2) wear versus time decreased following a logarithmic behavior. In cases of complete rehabilitation, load bearing CAD/CAM-composite restorations crowns should be critically considered for application due to their occlusal wear behavior.
